# Identifying and Manipulating Giant Vesicles: Review of Recent Approaches

**DOI:** 10.3390/mi13050644

**Published:** 2022-04-19

**Authors:** Taro Toyota, Yiting Zhang

**Affiliations:** 1 Department of Basic Science, Graduate School of Arts and Sciences, The University of Tokyo, 3-8-1 Komaba, Meguro-ku, Tokyo 153-8902, Japan; zy-si@g.ecc.u-tokyo.ac.jp; 2 Universal Biology Institute, The University of Tokyo, 3-8-1 Komaba, Meguro-ku, Tokyo 153-8902, Japan

**Keywords:** giant vesicle, micromanipulation, microscopy, microfluidic device

## Abstract

Giant vesicles (GVs) are closed bilayer membranes that primarily comprise amphiphiles with diameters of more than 1 μm. Compared with regular vesicles (several tens of nanometers in size), GVs are of greater scientific interest as model cell membranes and protocells because of their structure and size, which are similar to those of biological systems. Biopolymers and nano-/microparticles can be encapsulated in GVs at high concentrations, and their application as artificial cell bodies has piqued interest. It is essential to develop methods for investigating and manipulating the properties of GVs toward engineering applications. In this review, we discuss current improvements in microscopy, micromanipulation, and microfabrication technologies for progress in GV identification and engineering tools. Combined with the advancement of GV preparation technologies, these technological advancements can aid the development of artificial cell systems such as alternative tissues and GV-based chemical signal processing systems.

## 1. Introduction

Chemical sciences that mimic biological processes aim to improve our understanding of the exceptional molecular recognition and efficient molecular conversion capabilities of proteins, in addition to the mechanism of information conversion achieved by DNA and RNA. Supramolecular and systems chemistry [1] has led to substantial advancements in these studies. Artificial cells based on cell-sized molecular aggregates [2] have been actively produced in recent years to imitate cellular functions. 

Giant vesicles (GVs) have drawn attention as one of the cell-sized molecular self-assemblies [3]. GV is a molecular self-assembly composed of amphiphilic molecules forming a closed bilayer membrane in which the hydrophobic parts face each other in water and has a size greater than or equal to 1 μm. The self-assembled bilayer and cell membranes have equivalent main constituents, the phospholipids. Because of this, GVs have attracted scientific attention as a chemical model of cells.

**Figure 1:**
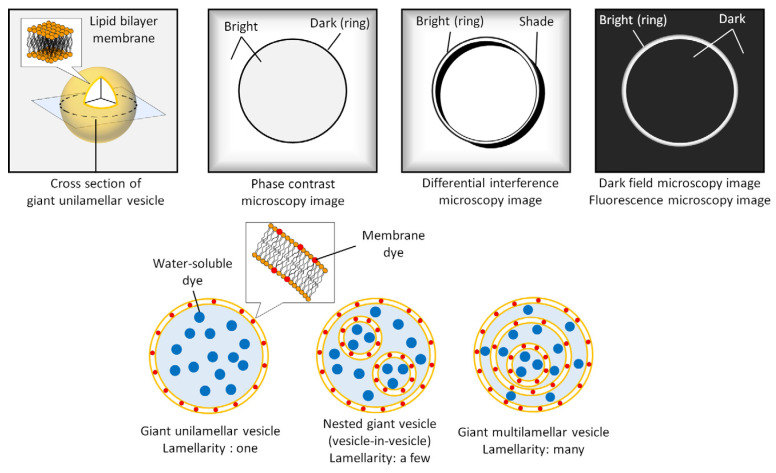
Schematic illustration of giant vesicles and their cross-sectional images in different kinds of microscopic technologies.

When vesicles are composed of phospholipids, which are the primary components of the cell membrane, they are labeled liposomes [4]. Vesicles that comprise synthesized amphiphilic macromolecules are called polymersomes [5]. Some papers describe liposomes and polymersomes with diameters of 1 μm or more as “giant liposomes” and “giant polymersomes”, respectively [6,7]. Moreover, based on structural appearance (Figure 1), GVs have been categorized as giant unilamellar vesicles (GUVs) made of a single bilayer membrane and giant multilamellar vesicles (GMVs) that have a nested structure or multiple membranes. Benefiting from the development of microfabrication technologies, GV preparation methods have made great strides in the last 20 years [3]. In particular, because GUVs are more likely to encapsulate biopolymers and nano/microparticles than GMV [8], many studies have been conducted on supramolecular chemistry for understanding cellular dynamics and functions using GUVs. For example, GUV-based sensors, processors, and actuators equipped with proteins, DNA, and RNA, have been invented [9,10,11,12,13]. Moreover, GVs that can cause proliferation of the membrane itself upon membrane precursors have also been reported [14,15,16,17,18].

To increase the level of GV functions aimed at the hierarchy of cellular functions, it is important to increase the structural hierarchy of GVs. The assembly of GVs has attracted attention as a basis for highly functionalized supramolecular chemical systems. In assembling technology for GVs, it is indispensable to determine the kind of GV to be assembled and identify each of the GVs. Therefore, in this review, we introduce the recent identification and characterization methods of GV and the progress of the manipulation methods developed by microfabrication technology. In addition, we discuss the process of constructing GV-based cell mimicry devices with higher-order functions.

## 2. Identification Methods of GVs

### 2.1. Morphology

GVs have diameters of 1 μm or greater and can be observed in situ without staining or fixing under an optical microscope due to the difference between the refractive index of the membrane and that of water. Conventional research on GVs has aimed to clarify the mechanism of cell shape using GVs as cell models, and thus, microscopic observation has been an important identification method for GV morphology (Figure 1). For example, a nested giant vesicle is characterized as a “vesicles-in-vesicle” configuration like eucaryotic cells. Individual observations of GVs are mostly performed by using phase-contrast, dark-field, or differential interference contrast microscopy. A phase-contrast microscope is used to observe GVs with a small number of vesicular membranes, resulting in fewer halos in the microscopy images. Dark-field microscopy completely shuts out the transmitted light around the vesicular membrane. As a result, it is possible to observe a single vesicular membrane with a thickness of approximately 5 nm. In contrast, differential interference microscopy has the advantage of not only observing GUVs, but also GMVs. The stained GVs are similarly observed under a bright-field microscope. GVs can encapsulate water-soluble dyes and colored particles that can be observed under a bright-field microscope. However, it is difficult to observe the vesicular membrane with ordinary hydrophobic dyes because of the interference of ambient transmitted light. Therefore, fluorescence microscopy, in which the ambient transmitted light is completely shut out, is a powerful tool for observing GVs. Additionally, confocal laser scanning fluorescence microscopy provides higher spatial resolution than epifluorescence microscopy and has become popular for GV observation among researchers. An automated algorithm for GV structural assessment using confocal laser scanning fluorescence microscopy images was developed by García-Sáez’s group [19]. More recently, Dimova’s group developed observation techniques for GUVs based on stimulated emission depletion (STED) microscopy, which can provide a high spatial resolution beyond the diffraction limit of light [20,21]. They found that the deduced membrane spontaneous curvature of nanometer-sized vesicular tubes produced from GVs, was in excellent agreement with theoretical predictions.

Although detailed optical microscopy observation of individual GVs is effective for scrutinizing the GV structural morphology, it is difficult to statistically and quantitatively evaluate the morphologies of large amounts of GV. Flow cytometry has been used to measure the GV population. While Vorauer-Uhl et al. first reported the flow cytometric evaluation of the size distribution of nanometer-sized fluorescence-labeled vesicles [22], Yomo’s group investigated the structural properties of large amounts of GVs [23]. The group used fluorescent proteins, such as green fluorescent protein, to evaluate the inner water volume of individual GVs. Fluorescence-labeled phospholipids were used to determine the lipid membrane volume via the thickness and surface area of the phospholipids. Additionally, they reported a structural comparison of GVs prepared using different methods [24,25]. It was found that the water-oil-emulsion templating method [26] for the preparation of GUVs can provide a large number of GVs with apparently unilamellar vesicular membranes, where the nominal number of bilayer membranes contained in the individual GV was found to be 2–3.

### 2.2. Lamellarity

Lamellarity of GV is defined as the number of bilayer membranes contained in individual GVs. The film swelling method, which is a standard preparation method for GVs, usually affords a mixture of GUVs (with a lamellarity of 1) and GMVs (with a maximum lamellarity of several thousand). The recent development of GV preparation methods, including the water-oil-emulsion templating method, enables GUV production and the encapsulation of water-soluble molecules and particles into GUVs [3]. A membrane permeation experiment using a membrane-penetrating protein, α-hemolysin [27], has become the standard method for determining whether GV observed under the microscope has a lamellarity of one [28]. α-Hemolysin is a channel-type membrane protein and composed of a hydrophobic part that recognizes and enters the lipid bilayer membrane and a hydrophilic part of several nanometers. When added to GVs, α-hemolysin pierces the outermost vesicular membrane from the outside and does not move into the interior. Therefore, if a water-soluble fluorescent molecule is encapsulated inside the GV and its lamellarity is one, the encapsulated molecule permeates and diffuses to the outside of the GV through the pores of the α-hemolysin. Recently, de novo designed transmembrane pores of proteins or DNA origami have also been reported by several groups [29,30]. 

A quantitative attempt to evaluate GV lamellarity was recently performed by Ishiwata’s group [31]. The group carefully observed individual GVs labeled by fluorescent phospholipids (the number of assessed GVs was over 200) and plotted the mean fluorescence intensities of GVs along the circumference against the GV diameter. They found that the plot showed discrete distributions and could be classified into several groups. The GV group with fluorescence intensities similar to the predicted value of the ideal GUV and few dependencies on size were assigned to the GUV. They found that several fluorescent dyes for the vesicular membrane of GVs could affect the lamellarity of GVs produced by the water-in-oil emulsion templating method. By fitting a model to differential interference contrast microscopy images, McPhee et al. measured the lamellarity of GVs and validated the data by comparison with epifluorescence microscopy images [32]. This approach is important in terms of label-free measurements, which excludes the inherent drawbacks of fluorescent labeling such as sample modification and photobleaching.

### 2.3. Membrane Bending Rigidity

The membrane bending rigidity or membrane modulus of GV is one of the most important characteristic parameters that have been measured historically in many studies from the biophysical viewpoint of cell membranes. Many measurement studies have been conducted to observe vesicular membrane deformation caused by the suction of a glass capillary (micropipette) under a microscope [33]. The micropipette assay of GVs also enables the measurement of the viscosity of the vesicular membrane by oscillatory shear gradient [34]. A microfluidic device that can fold and aspirate a GV in a narrow space, called an on-chip micropipette, has been developed [35]. Recently, an electroporation technique was applied for membrane tension measurement as well as line tension measurement because GV deformation and rupture are regulated by electroporation conditions [36]. However, the micropipette is brought into contact with a GV and a direct force is applied as a perturbation to the vesicular membrane; thus, physical interference has sometimes been regarded as a perturbation (plastic deformation) in these methods [37]. Therefore, as a non-contact and non-perturbative measurement, observing a wavy motion mode, called fluctuation, has been conducted. This fluctuation of the GV membrane occurs due to the random collision of water molecules and is used to calculate the membrane bending rigidity [38,39,40]. After the fluctuations of the GV membrane around an equilibrium shape are monitored, Fourier decomposition of the contours is applied, and the mean square values of the shape deviations are determined.

**Figure 2:**
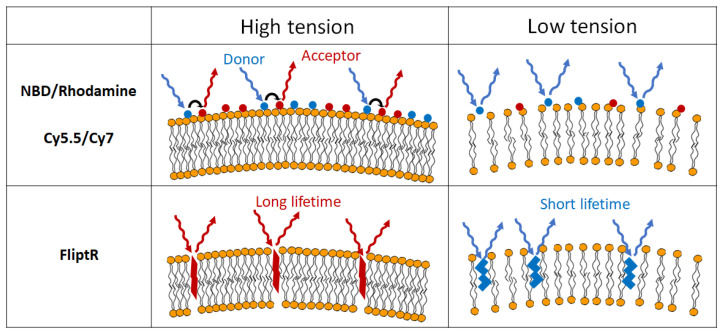
Schematic illustration of visualizing membrane tension by fluorescent membrane dyes.

In addition, visualization of membrane bending rigidity using fluorescent dyes for GV membranes has been developed (Figure 2). A specific pair of fluorescent molecules can change their fluorescence intensity through Förster resonance energy transfer (or fluorescence resonance energy transfer), which reflects the distance between the molecules shifted by bending the vesicular membrane [41]. Recently, Roux’s group observed that a hydrophobic dithienothiophene derivative, FliptR (fluorescent lipid tension reporter), buried in the vesicular membrane, can change excitation maxima and fluorescence lifetime responding to the membrane tension and rigidity [42]. The group successfully visualized not only GV membrane tension but also that of cell membranes using FliptR.

### 2.4. Permeability and Intramembrane Ion Current

As explained previously, the membranes of GVs are semipermeable, that is, water-soluble molecules with large molecular weights and ions scarcely permeate the membrane, while water molecules can permeate. Membrane permeability is another important physical parameter for measuring GVs through cell membranes. A single GUV observation for membrane permeation of encapsulated water-soluble fluorescence dyes under a microscope is repeatedly and quantitatively performed to determine the reliability of the permeation rate. Yamazaki’s group applied this method to clarify the mechanism of bioactive substances, such as magainin 2, which interacts with cell membranes to enhance membrane permeation upon chemical stimulus of such substances [43]. The apparatus for membrane permeation measurement was equipped with a glass capillary that gently added a substance in the vicinity of the GVs. In recent years, after trapping GVs in cup-shaped microstructures fabricated in microfluidic devices, researchers have examined GV membrane permeation under a fluorescence microscope upon exposure to bioactive substances such as α-hemolysin. [44]. Sugiyama et al. recently reported that exposure to water-soluble fluorescent molecules such as uranine, results in their accumulation inside GVs against their concentration gradient under a steady flow [45,46]. Their evaluation of this unusual phenomenon indicated the possibility of asymmetric distribution of phospholipids between the inner and outer leaflets of GVs when the GVs are exposed to a steady flow. 

The ion current of the GV membrane has drawn attention in the construction of model nerve cells based on GVs. The physiological measurement of ion currents in the nerve cell membrane, called the patch-clamp technique, also enables us to measure those in the GV membrane. Hamada et al. and Fukuda et al. independently applied the whole-cell patch-clamp technique to measure the ion currents of GV membranes inserted with membrane proteins [47,48]. Velasco-Olmo et al. reported that the whole-cell patch-clamp method can be applied for the lamellarity assessment of GV membranes generated on spherical objects [49].

### 2.5. Other Properties

Other properties of vesicles, such as the zeta potential and heat of transition of the vesicular membrane, are significant physical properties in terms of not only the model cell membrane, but also the drug delivery carrier. These properties have been measured for nanometer-sized vesicles because such vesicles, called small or large vesicles (<1000 nm), can be uniform through ultrasonication and filtration [50,51]. However, to the best of our knowledge, there is no report on a methodology that enables the measurement of the zeta potential or the heat of transition of individual GV. One reason for this is that conventional preparation methods for GVs are not optimized to produce GVs with uniform sizes, structures, and properties. Recent advances in GV preparation methods to provide GUVs of uniform size [52] will solve this problem. Another solution is the development of a micro-apparatus for electrophoretic mobility and calorimetric measurements using microelectrodes and thermocouples that can stably sweep the electric field. Because such microfabricated apparatus has already been applied to measure the properties of single cells [53], they will be used to single GV measurements.

## 3. Manipulation Methods of GVs

### 3.1. Immobilization on a Substrate

The more detailed the observation of each GV under a high-resolution optical microscope, the longer the observation time. This sometimes causes difficulties in capturing 3D images of individual GVs owing to Brownian motion or membrane fluctuation. The current technical problem has been solved through the development of high-speed scanning confocal laser fluorescence microscopy [54] and light-sheet fluorescence microscopy [55]. However, the importance of establishing a method that can immobilize GVs on a substrate for detailed observation has increased.

A commonly used method is to modify GVs with biotin-tagged phospholipids. This results in GVs that are heavier than their surroundings, which settle onto a substrate coated with avidin. For example, encapsulation of sucrose inside GVs and addition of same osmolarity glucose on the outside [56]. In contrast, Cuvelier et al. reported avidin-coated GVs immobilized on a substrate modified with biotin-tagged casein [57]. They observed a GV response under shear stress in the flow field, due to the strong interaction of biotin-avidin bonds. In addition to biotin-avidin interaction, there were reports that the linker between GVs and the substrate is DNA [58] or protein (integrin) [59]. Because the bilayer membrane is likely to get wet on a flat substrate, it is necessary to pay attention to the practice of GV immobilization. The settled GVs can rupture on the substrate and transform into a bilayer membrane film [60]. To avoid such rupture, GUVs can be buried in an agarose gel at concentrations of 0.25–0.5% *w*/*v*. Lira et al. observed that the lateral displacement of GUVs in agarose gels was completely suppressed [61].

### 3.2. Size Sorting and Purification

The preparative technology for GVs has advanced not only in controlling lamellarity but also in fabricating uniform sizes [52,62,63]. In some devices, there are constraints on the lipid composition, applicable buffered solution, and flow field; as a result, it is not always possible to prepare monodispersed GVs under general-purpose experimental conditions. Therefore, size-sorting methods for GVs of various sizes are desirable. Several groups have reported GV size sorting (called purification) using filters [64,65,66]. All groups filtered the GV dispersion using membrane filters made of polycarbonate, where the pores size ranged from 3 μm to 12 μm. Each group remarked that gentle extrusion is important for the purification process. 

**Figure 3:**
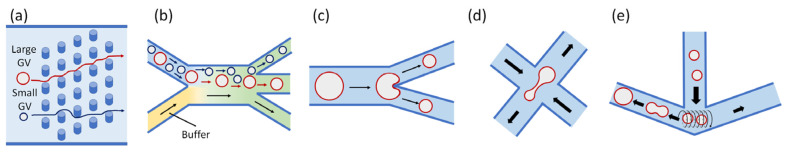
Schematic illustrations of microfluidic devices for size sorting, purification, and manipulation: (**a**) deterministic lateral displacement, (**b**) pinched-flow fractionation, (**c**) Y-junction for splitting GVs, (**d**) cross-road for holding and stretching GVs, and (**e**) anchor-shape junction for vesicle fusion.

In addition, size sorting techniques for GVs using microfluidic devices have recently been proposed following cell sorting microfluidic technology [67]. Kazayama et al. reported that deterministic lateral displacement (DLD) is a promising mechanism for GV size sorting (Figure 3a). This is carried out by evaluating the DLD for GV dispersion with a microfluidic device and integrating the DLD and region of trapping microstructures [68]. They achieved monodisperse trapped GVs with a variation coefficient of approximately 10%. Robinson’s group also suggested a size-sorting microfluidic mechanism with massive trapping of GVs by devising a trapping structure [69]. Recently, using the stream bifurcation structure of a microfluidic device, GV purification, which separates GUVs from a mixture of GUVs and additives (including nanometer-sized particles), was performed by a pinched-flow fractionation mechanism (Figure 3b) [70].

### 3.3. Microfluidic Manipulation

The trapping and morphological change induction of GVs by hydrodynamic fields have become popular in microfluidic device development as well as the GV preparation method. As already mentioned, GVs are likely to be captured by simply pouring the GV dispersion into a microfluidic device equipped with cup-shaped microstructures with a narrow gap. Ditrrich’s group developed a device that created a curtain of air bubbles around each GV-trapping microstructure to protect the trapped GV from the surrounding flow field [44]. Yamada et al. reported that GV can be trapped and purified by a well-type space with a gap in the vertical direction [71].

The advantage of using microfluidic devices is that, owing to the development of materials and hydrodynamic methodologies for microfluidic devices, it is possible to freely design shapes and pour GV dispersions for observation and manipulation. For instance, by devising the shape of the trapping structure, Schwille’s group held to trap the prolate-shaped GVs and found a novel pattern formation of the encapsulated protein, FtsZ, that self-assembles in response to the GV shape [72]. Dekker’s group developed a microfluidic device that could induce GV splitting (Figure 3c) [73]. Kumar et al. manufactured a crossroad-type microfluidic device to trap and deform GV in the center (Figure 3d) [74]. Stone’s group developed a microfluidic device that causes a vortex at a junction and found that vesicle fusion occurs there (Figure 3e) [75]. Lira et al. observed the binding and fusion of nanometer-sized vesicles on GV membranes with different surface charges [76]. Sen’s group reported microfluidic devices that can evaluate liposomes propelled by enzymatic reactions on liposomal surfaces [77]. These innovations will continue to stimulate the development of artificial cell systems.

### 3.4. Micropipette Manipulation

**Figure 4:**
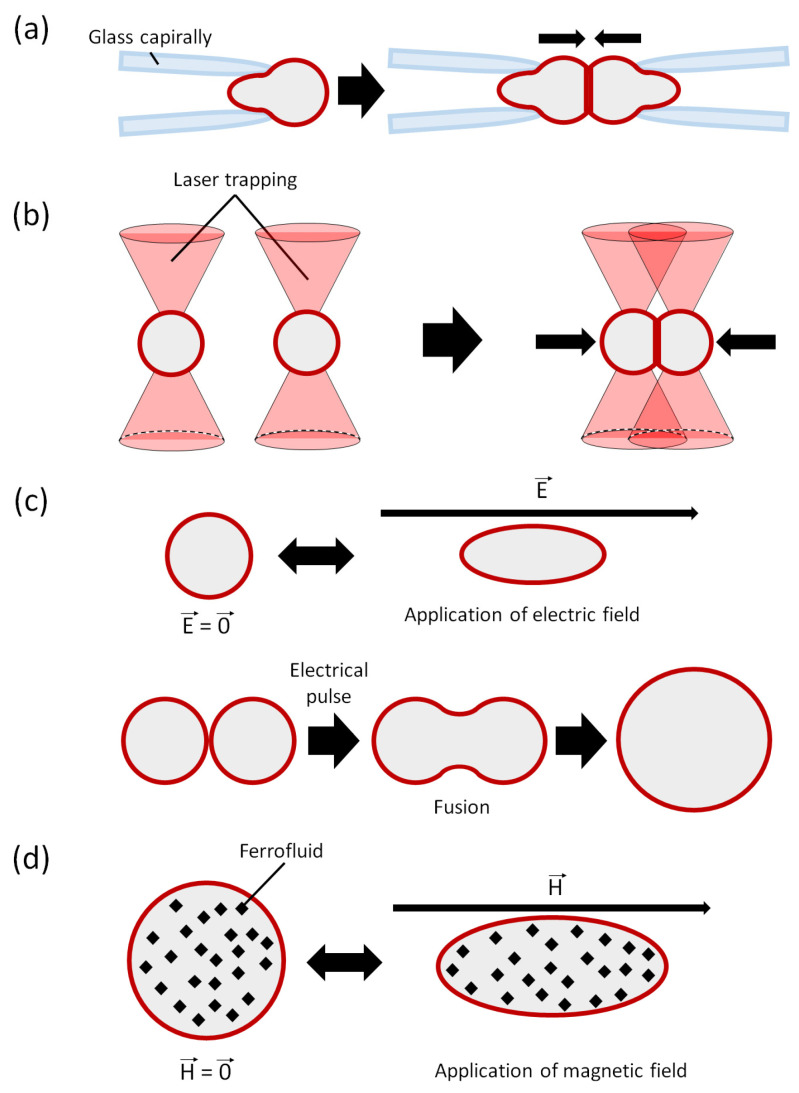
Schematic illustration of manipulation of GVs by (**a**) glass capillary (micropipette), (**b**) optical tweezers, (**c**) electric field, and (**d**) magnetic field (for GVs encapsulating ferrofluid).

As mentioned above, measurement of the membrane bending rigidity of GVs is conventionally performed using a glass capillary as a micropipette. Micropipette aspiration can hold them and allow morphological changes in GVs. When the tip of the micropipette is sufficiently narrower than the GV and the suction pressure is sufficiently strong, the GV membrane stretches to form a tubular structure [78]. Furthermore, in two micropipette systems that enable the holding of one GV at each tip of the micropipettes, the adhesion between the two GVs can be evaluated by bringing the GVs close to each other via micropipette manipulation (Figure 4a).

Orwar’s group extended the micropipette technique to morphological changes in GVs [79]. The group established a glass capillary with a microelectrode inside, which is often used in the patch-clamp technique to measure intramembrane ion currents. They placed the glass capillary near a GV, together with the counter electrode and found that when an electric field was applied, part of the GV membrane could be stretched and transformed into a tubular structure, and then attached to the tip of the glass capillary. They showed that by adjusting the electric field voltage, the tip of the tubular membrane is led to adhered to an adjacent GV, and the GVs were connected to exchange the internal aqueous solution [80]. In contrast, Lobovkina’s group reported that by applying positive pressure toward the inside of the GUV at the contact tip of a micropipette, a string-bead structure made of a stretched GV membrane was produced inside the GUV [81]. In addition, microinjection is also possible with a micropipette by piercing a part of the GV membrane with a tip with a diameter of 1 μm [82,83,84].

### 3.5. Optical Trapping and Acoustic Trapping

Laser technology has advanced research in the scientific and engineering fields, and the optical tweezers (or laser trapping) have had a great impact on GV manipulation. In the initial research on optical tweezers on GVs, the transformation of GVs was observed due to the light radiation pressure of a laser applying a GV membrane, which has a higher refractive index than water [85,86]. GVs encapsulated in an inner aqueous solution with a high refractive index can be carried by moving the stage while the GVs are trapped or by moving the laser focus [87,88]. When a microbead is held by an optical tweezer and brought into contact with the GV membrane, a nanometer-sized tubular structure is formed by pulling the microbead [21]. Inaba et al.examined membrane stretching using microbeads that were suspended and shifted using an optical tweezer inside of a GV [89]. Taking advantage of the fact that optical tweezers exert various effects on GVs, Elani’s group successfully collected multiple GUVs with optical tweezers, brought them into contact with each other, and further pierced them to execute a sequential enzymatic reaction inside the partially fused GVs (Figure 4b) [90]. Shiomi et al. developed a platform that can repeat the fusion and division of GVs using a combination of optical tweezers, electroporation, and temperature stimulation [91]. Recently, Yoshino et al. showed that a sub-micrometer-sized vesicle could be captured and transported by a gradient of the force of light evanescent waves along an optical nanofiber [92].

GV manipulation is also realized using sound pressure. The advantage of sound waves is irradiation variation, that is, squeezing sound pressure in a relatively small area or creating a periodic pattern of sound pressure in a limited space. In the former case, pore formation in the GV membrane occurs [93]. In the latter case, different types of GVs in a mixed dispersion can be sorted by composition, owing to the difference in the sound pressure response [94]. A collaboration between Mann and Drinkwater’s group revealed that GVs gathered and were forced to deform under an acoustic pressure field [95,96]. Interestingly, Pereno et al. revealed that a vortex stream occurs inside GUVs when they are exposed to ultrasound pressure [97].

### 3.6. Electric and Magnetic Field Application

The electric and magnetic fields directly affect the properties of the lipid molecules, so that GV manipulation, including shape change of GVs, is easily performed (Figure 4c). The electroporation of GV is the most popular phenomenon, as mentioned above. Moreover, by arranging the application condition of voltages, it is possible to keep GVs with a hole of several micrometers in diameter or to keep them in an angular shape that is not observed in the equilibrium state [98,99,100]. In contrast, dielectrophoresis leads to GVs gathering; hence, GVs can be fused by controlling the application of an electric field [101,102]. Nomura and Morishima’s group have independently reported that GV was fused with cells by dielectrophoresis [103,104]. By preparing and mixing two types of GVs with different dielectrophoresis responses and applying an alternating electric field to the mixture dispersion, the frequency shift for dielectrophoresis successfully sorted them into two groups [105]. When GUVs are irradiated by microwaves, which are electromagnetic waves, the difference in their microwave transmittance depends on the membrane composition [106]. This approach is expected to be a new non-contact GV manipulation technique.

GVs respond to a magnetic field that is a result of diamagnetic susceptibility of the lipid bilayer membrane. Historically, Helfrich’s first report on GV morphology suggested the perturbation of GV shapes by a magnetic field [107]. Recently reports showed the fusion and fission of vesicles were induced by strong magnetic field application [108]. Suzuki et al. reported that tubular GVs composed of phospholipids and collagen, which have the anisotropic diamagnetic susceptibility different from that of phospholipid membranes, form a specific curved structure (called elastica) in response to a high magnetic field [109]. In contrast, Bacri et al. showed that GVs containing superparamagnetic nanoparticles, that is, ferrofluid, are deformed by a weak magnetic field (Figure 4d) [110]. Liposomes encapsulating such nanoparticles are called magnetoliposomes and have drawn considerable attention as novel drug delivery carriers [111]. Nomura’s group reported that cell-sized magnetoliposomes can be remotely controlled and manipulated by an external magnetic field [112]. Han’s group demonstrated the assembly of magnetoliposomes by an external magnetic field. They also observed that chemical reactions of magnetoliposomes, including glucose oxidase and a transmembrane pore peptide, melittin, which can produce H_2_O_2_ inducing cell death in the vicinity of the assembly (GUV colony) under glucose exposure [113].

### 3.7. Laser-Assisted Three-Dimensional (3D) Printing Inside of GUV

3D printing technology has sparked a revolution in the micro-construction of molds for fundamental material research and industrial applications [114]. One of the most eye-catching features of this technology is that 3D printing is based on digital fabrication technologies. Thus, through computational design, the construction of molecular aggregate in a 3D manner is practically and reproducibly controlled. Göpfrich’s group developed a two-photon laser-assisted 3D printing technology inside GUVs [115,116]. They constructed tubular 3D molds not only inside but also in the intramembrane of GUV and the membrane permeability of the GUV was changed by the mold. This emerging technique opens future manufacturing and tuning of artificial cells.

## 4. Summary

In this paper, we review recent developments in GV identification and manipulation methods and summarize the advantages of basic techniques for assembling GVs as building blocks. Several approaches overlap with cell manipulation techniques. However, GVs have no cytoskeleton or cell wall and therefore require careful handling of their physical properties. Imminent enhanced development of artificial cell construction is expected due to these methods, leading to the construction of elaborate artificial tissues and information processing systems composed of GVs [95,113,117,118].
